# Pathways to eating disorder care: A European multicenter study

**DOI:** 10.1192/j.eurpsy.2023.23

**Published:** 2023-04-24

**Authors:** Alessio Maria Monteleone, Eugenia Barone, Giammarco Cascino, Ulrike Schmidt, Philip Gorwood, Umberto Volpe, Giovanni Abbate-Daga, Giovanni Castellini, Marina Díaz Marsá, Angela Favaro, Akira Fukutomi, Sebastien Guillaume, Petr Minařík, José Antonio Soriano Pacheco, Matteo Panero, Hana Papežová, Valdo Ricca, Cristina Segura-Garcia, Elisabetta Scanferla, Marta Tyszkiewicz-Nwafor, Fernando Fernandez-Aranda, Ulrich Voderholzer, Janet Treasure, Palmiero Monteleone

**Affiliations:** 1 Department of Psychiatry, University of Campania L. Vanvitelli, Naples, Italy; 2 Department of Medicine, Surgery and Dentistry ‘Scuola Medica Salernitana’, University of Salerno, Salerno, Italy; 3 King’s College London, Department of Psychological Medicine, Institute of Psychiatry, Psychology and Neuroscience, London, UK; 4 South London and Maudsley NHS Foundation Trust, London, UK; 5 Université Paris Cité, Paris, France; 6 GHU Paris Psychiatrie et Neurosciences (CMME), Paris, France; 7 INSERM U1266, Paris, France; 8 Section of Clinical Psychiatry, Department of Clinical Neurosciences/DIMSC, Università Politecnica delle Marche, Ancona, Italy; 9 Eating Disorders Center for Treatment and Research, Department of Neuroscience, University of Turin, Turin, Italy; 10 Psychiatry Unit, Department of Health Sciences, University of Florence, Florence, Italy; 11 Eating Disorder Unit, Hospital Clínico San Carlos, Universidad Complutense, Cibersam, Madrid, Spain; 12 Neurosciences Department, University of Padua, Padua, Italy; 13 Department of Emergency Psychiatry and Acute Care, CHU Montpellier and University of Montpellier, Montpellier, France; 14 First Medical Faculty of Charles University and General University Hospital in Prague, Prague, Czech Republic; 15 Servei de Psiquiatría, Hospital de Sant Pau, Barcelone, Spain; 16 Department of Medical and Surgical Sciences, University Magna Graecia of Catanzaro, Catanzaro, Italy; 17 Department of Psychiatry, Poznan University of Medical Sciences, Poznan, Poland; 18 Clinical Psychology Unit, Bellvitge University Hospital-IDIBELL and CIBERobn, ISCIII, Barcelona, Spain; 19 Clinical Sciences Department, School of Medicine and Health Sciences, University of Barcelona, Spain; 20 Schoen Clinic Roseneck, Prien am Chiemsee, Germany; 21 Department of Psychiatry and Psychotherapy, University Hospital Munich, Munich, Germany

**Keywords:** Barriers, eating disorders, educational, health care policy, pathways to care

## Abstract

**Background:**

The aim of this study was to assess barriers and facilitators in the pathways toward specialist care for eating disorders (EDs).

**Methods:**

Eleven ED services located in seven European countries recruited patients with an ED. Clinicians administered an adapted version of the World Health Organization “Encounter Form,” a standardized tool to assess the pathways to care. The unadjusted overall time needed to access the ED unit was described using the Kaplan–Meier curve.

**Results:**

Four-hundred-nine patients were recruited. The median time between the onset of the current ED episode and the access to a specialized ED care was 2 years. Most of the participants did not directly access the specialist ED unit: primary “points of access” to care were mental health professionals and general practitioners. The involvement of different health professionals in the pathway, seeking help for general psychiatric symptoms, and lack of support from family members were associated with delayed access to ED units.

**Conclusions:**

Educational programs aiming to promote early diagnosis and treatment for EDs should pay particular attention to general practitioners, in addition to mental health professionals, and family members to increase awareness of these illnesses and of their treatment initiation process.

## Introduction

Eating disorders (EDs) are severe mental illnesses with modest rates of remission and frequent relapses [1–3]. It is widely known that their early detection and treatment lead to a better outcome [4–6] and reduced social burden [7]. The National Institute for Health and Care Excellence (NICE) guidelines [8] recommended that treatment should be provided “at the earliest opportunity.” However, literature studies showed a delay in the access to specialist treatments for patients with either anorexia nervosa (AN) [9] or other EDs [10] that has been estimated between 2 and 5 years. Illness-related factors, such as the lack of patients’ awareness of illness, or the stigma related to mental illnesses, contribute to this delay[11]. Several barriers to treatment access also occur and may depend on the characteristics of the clinical pathways that lead individuals with EDs to specialist care although they have not been adequately investigated [11, 12]. Indeed, most of the studies adopted a qualitative method, which did not allow to quantify the importance of each barrier and did not employ validated questionnaires assessing the relative contribution of each barrier [11].

The complexity of ED pathways is amplified by the challenging nature of these illnesses, which are associated with high rates of psychiatric [13, 14] and somatic comorbidities [15] and a high mortality risk [16, 17]. Thus, their treatment typically involves several healthcare disciplines (i.e., internal physicians, psychologists, and nutritionists) [18, 19]. This explains why individuals with EDs make considerable use of mental and physical health services [20] and consult general practitioners more frequently than controls in the five years before their ED diagnosis [21]. A recent study [22] showed that nearly all patients with AN or bulimia nervosa (BN) in Taiwan had sought help for physical problems in the year before the ED diagnosis: in this period, medical wards were more often involved in the hospitalization of these patients than psychiatric wards and gynecologists and family medicine were the most consulted specialists [22]. Furthermore, the leading diagnoses were not ED diagnoses (instead, anxiety and depressive disorders and personality/neurotic/sleep disorders were diagnosed) or were broadly defined EDs [22, 23]. In sum, the detection of other leading diagnoses is a diagnostic delay that contributes to lack of early referral to treatment, and optimizing early detection and access to specialist services should be clinical and research priorities [24].

A useful tool to explore the clinical pathways of patients with EDs to specialist services is the “pathways to care” approach. The “pathways to care” studies have been widely used to explore the help-seeking behaviors of individuals suffering from severe illnesses [25, 26]. More recently, a specific and standardized tool has been developed in collaboration with the World Health Organization to assess the routes followed by psychiatric patients to seek help for their mental health problems [27]. This instrument allowed researchers to assess variations of pathways to mental healthcare across different countries [28].

The aim of this multicenter study was to assess the pathways to specialist care in individuals suffering from EDs in different European countries. First, the number and the type of health professionals involved in the pathway, who suggest seeking care, and the symptoms occurring before the referral to specialist health professionals have been identified. Second, the length of the pathway occurring between the onset of the current ED episode or the access to the first health professional of the pathway up to the referral to specialist ED services has been explored. Third, the barriers interfering with the referral to specialist ED centers have been investigated. Based on previous findings in the Italian population [29], we hypothesized that the involvement in the pathways of non-ED specialist healthcare professionals may promote delayed referral to specialist ED services, while the suggestion to seek care coming from relatives may favor earlier referrals. These findings will help to extend previous evidence [29] and to develop international educational programs.

## Methods

### Participants

Patients were recruited from those consecutively seeking care at the following European specialist ED units: University of Campania Luigi Vanvitelli (Italy); General Hospital and 1st Medical Faculty of Charles University in Prague (Czech Republic); CMME, GHU Paris Psychiatrie et neurosciences (France); Université de Montpellier (France); Schoen Clinic Roseneck (Germany); Univeristy of Florence (Italy); University of Padua (Italy); University “Magna Græcia” of Catanzaro (Italy); University of Turin (Italy); Poznan University of Medical Sciences (Poland); Hospital Clínico San Carlos, Universidad Complutense (Spain); Servei de Psiquiatría, Hospital de Sant Pau, Barcelona (Spain); South London and Maudsley NHS Foundation Trust Eating Disorders Service, London (United Kingdom). A specialist ED unit was defined as a center providing a comprehensive multidisciplinary treatment by professionals with long-lasting experience in the treatment of EDs. An invitation to participate in the study was sent to all members of the ED sections of the European Psychiatric Association and of the World Psychiatric Association. Twenty-three members responded and were affiliated with a specialist ED unit meeting the above criteria and were included among the recruiting centers. Twelve out of 23 centers were not able to carry out the survey mainly due to internal technical difficulties (namely, lack of available research staff).

In accordance with the pathways methods [30], one Principal Investigator (PI) was identified in each ED Unit who had to recruit at least 25 patients who had access to the specialistic ED center (inpatient, outpatient, or day-patient). The recruitment was scheduled from September 1, 2020 to October 31, 2020: with respect to usual pathways studies [30, 31] the survey period was extended from one to two working months to ensure each center to collect at least 25 patients. However, the COVID-19 pandemic restrictions limited the access to care all over the world also for individuals with EDs [32]. Therefore, the deadline for recruitment was extended to September 2021 to allow each participating center to enroll patients in a period in which the access to specialist care was not affected by the local restrictions imposed for the pandemic. If a center had the possibility to recruit more than 25 patients in the study period, this was allowed. The criteria for patient inclusion were: a) diagnosis of ED according to the DSM-5 criteria [33], confirmed by the Structured Clinical Interview for DSM-5 –Clinician Version (SCID-IP) [34]; b) acceptance to join the study. Exclusion criterion was the referral to one of the participating ED centers directly from another ED specialist center.

The study was approved by the Ethical Committee of the Coordinating Center (i.e., the University of Campania “L. Vanvitelli,” Naples, Italy, number of protocol: 0015734/i 01/07/2020). Each participant was asked to give his/her written informed consent to participate into the study after being properly informed with a complete description of the study aims and methods.

### Materials and procedure

**Figure 1.**
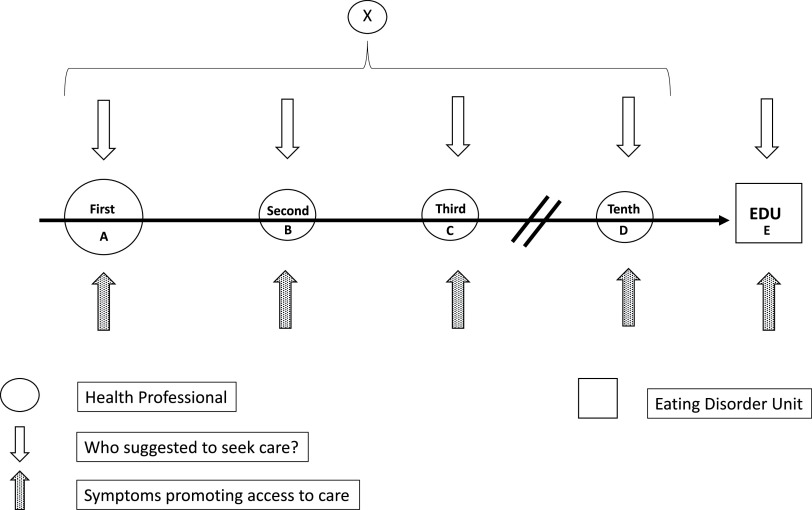
Diagram of the general pathway to care for participants. Point “x” indicates one of the health professionals who were seen before the access to the ED unit.

A pathway to care starts when a person develops a psychological problem, or a health problem with an important psychological component, and the first decision is taken to seek care from a health professional. Subsequently, any number of different (including mental) health professionals may be involved, but the pathway ends with the current consultation at the specialist ED unit. A diagram of the overall pathway to care for participants has been provided in Figure 1.

In order to define the pathways to care of individuals with EDs, a face-to-face structured interview (the “Encounter Form,” EF) [31] was employed. This is a slightly modified version of the original tool developed by the World Health Organization and has been described in the Supplementary material S1.

### Statistical analysis

Descriptive statistics were used to summarize study variables. The number of months elapsing between the onset of the symptoms of the current ED episode and the access to a specialist ED unit, the number of months elapsing between the first contact with a health professional of the specialist pathway and the arrival at the ED unit, and the number of health professionals included in the specialist pathway for ED care were reported as median values as the distribution of these variables was significantly skewed. The prevalence of each variable measured at the time of referral to the specialist pathway was compared with that measured at the referral to the specialist ED unit through McNemar’s test for non-parametric data. Differences among countries were calculated by means of chi-square test for categorical variables and one-way ANOVA followed by Tukey’s post-hoc test for continuous variables.

The unadjusted overall time to access to ED units was described using the Kaplan–Meier curve. Multivariate Cox proportional hazards regression was used to assess associations, measured as hazard ratios (HRs), between covariates and time [35, 36]. Patients’ age, marital status, country, social position, presence of ED specific symptoms or somatic symptoms or general psychopathology, current ED diagnosis, history of access at another ED unit, the previous carer, and who suggested the patient to seek help were the covariates entered in the model. In the “general psychopathology” variable anxious and depressive symptoms, interpersonal and behavioral problems, suicide attempts, and sleep disturbances were included in a unique category. In the “who suggested the patient to seek help” variable patient, family/partner, friends/workmates, health professionals (previous carer, nutritionist, psychologist, general practitioner) were included as categories. The time elapsing from the onset of the current ED episode to the access to the specialist ED unit was included as the outcome of the model.

Significance level was set at *p <*0.05. Analysis was performed using R, version 4.2 [37].

## Results

### Demographic characteristics of the sample

The final sample was composed of 409 individuals (383(94.3%) females, 26(5.7%) males) with a diagnosis of an ED (213(52.1%) with AN, 89(21.8%) with BN, 41(10%) with binge-ED, 66(16.1%) with other specified feeding or EDs). Mean age was 26.6 ± 11.2 years. Most of the participants were recruited in Italy (45%), were single (74%), on average social status (61%), Caucasian (86%), and with no history of previous care in a specialist ED unit (73%). The demographic and clinical characteristics of the study sample are reported in Table 1.

**Table 1.** Demographic and clinical characteristics of the sample.

	Number of participants	%
	409 (383 F, 26 M)	
Mean age (SD) – years	26.6 (11.2)	
Provenance		
Italy	185	45.4
Poland	25	6.1
France	67	16.3
Czech Republic	26	6.3
Spain	55	13.4
United Kingdom	25	6.1
Germany	25	6.1
Marital status		
Single	301	73.6
Engaged	39	9.5
Married	60	14.7
Separated	7	1.7
Other	2	0.5
Social position		
On average	250	61
Above average	70	17
Below average	59	14
Ethnicity		
Caucasian	352	86
Non-Caucasian	13	14
Past history of care in EDU		
Yes	299	73
No	104	25

### Characteristics of the pathways to care

The main variables characterizing the pathways to care are reported in Table 2. The median time elapsing between the onset of symptoms of the current ED episode and the access to a specialist ED unit was 2 years (Min=0; Max=36).

**Table 2.** Characteristics of the pathways to care.

		Median (Min;Max)
Months between the onset of current ED episode and access to ED unit		24 (0;36)
Length of the pathway (months)		24 (0;35)
Number of health professionals		2 (1;10)
	Number	Percent (%)
First health professional of the pathway		
Psychiatrist	107	26.2
General practitioner	101	24.7
Psychologist	70	17.1
Nutritionist	36	8.8
Hospital doctor	29	7.1
Endocrinologist	3	0.7
Pediatrician	23	5.6
Gynecologist	2	0.5
Occupational doctor	1	0.2
Other (non-specified)	6	1.5
None (direct access to ED unit)	31	7.6
Health professional who was seen before the access to ED unit		
Psychiatrist	140	34.2
General practitioner	89	21.8
Psychologist	70	17.1
Nutritionist	34	8.3
Hospital doctor	35	8.6
Nobody	21	5.1
Pediatrician	9	2.2
Other (non-specified)	8	2
Endocrinologist	2	0.5
Refused	1	0.2
Most common symptoms that promoted the referral to the first health professional of the pathway		
Eating-related symptom	256	68.4
Depressive symptom	104	27.9
Anxiety	85	22.7
Somatic symptom	53	14.2
Organic symptom	27	7.2
Interpersonal problems	27	7.2
Behavioral disorders	20	5.4
Sleep disturbance	18	4.8
Suicide attempt	8	2.1
Most common symptoms that promoted the arrival at the specialist ED unit		
Eating-related symptom	356	87.7
Depressive symptom	178	43.8
Anxiety	167	41.1
Somatic symptom	127	31.3
Sleep disturbance	77	19
Interpersonal problems	65	16
Organic symptoms	53	13.1
Behavioral disturbance	53	13.1
Suicide attempt	20	4.9
Psychotic symptoms	6	0.1
Suggestion to seek care that promoted the access to the specialist pathway		
Family	192	53.2
Patient	141	39.1
General practitioner	10	2.8
Friends	8	2.2
Partner	5	1.4
Psychologist	3	0.8
Workmate	1	0.3
Previous carer	1	0.3
Suggestion to seek care that promoted the arrival at the specialist ED unit		
Family	164	40.3
Patient	171	42
Previous carer	129	31.7
Friends	19	4.7
Partner	14	3.4
Workmates	4	1
Nutritionist	1	0.2
Psychologist	1	0.2
General practitioner	1	0.2
Psychiatrist	1	0,2

The average number of health professionals included in the specialist pathway for ED care was 2 (Min=0; Max=10); most of the participants (92%) did not directly access the specialist ED unit. The most frequent first health professionals on the pathway (Figure 1, point A) to specialist ED care were psychiatrists (26.2%), general practitioners (24.7%), and psychologists (17.1%). The psychiatrists were also the most prevalent (34.2%) health professionals who were seen before the access to the specialist ED unit (Figure 1, point X), followed by general practitioners (21.8%), psychologists (17.1%), nutritionists (8.5%) and hospital doctors (8.3%). The prevalence of general practitioners as the first health professional of the pathway (24.7%) was higher than that of general practitioners as the health professionals before the referral to the ED unit (21.8%) (*p =* 0.012).

The most common symptoms that promoted the referral to the first health professional of the pathway (Figure 1, point A) were eating-related symptoms (68.4%), followed by depressive (27.9%), anxiety (22.7%), and somatic (14.2%) symptoms. The most common symptoms that promoted being seen at the specialist ED unit (Figure 1, point E) were eating-related symptoms (87.7%), followed by depressive (43.8%), anxi (41.1%), and somatic (31.3%) symptoms. All these symptoms were more frequent at the referral to the specialist ED unit than at the referral to the first health professional of the pathway (all *p <*0.01).

The suggestion to seek care that promoted the access to the specialist pathway (Figure 1, point A) came from family (53.2%) or from patients themselves (39.1%). In the remaining cases (less than 10%) general practitioners, friends, partners, workmates or other health professionals suggested seeking care. The suggestion to seek care that promoted being seen at the specialist ED unit (Figure 1, point E) came from patients themselves (42%) or family (40.3%) in most of the cases: the involvement of the family was less frequent in comparison to that observed at the referral to the first health professional of the pathway (*p <*0.01). In the remaining cases, friends (4.7%) and partners (3.4%) were the most frequent categories in the suggestion to seek care at the ED unit.

### Differences among countries

**Figure 2.**
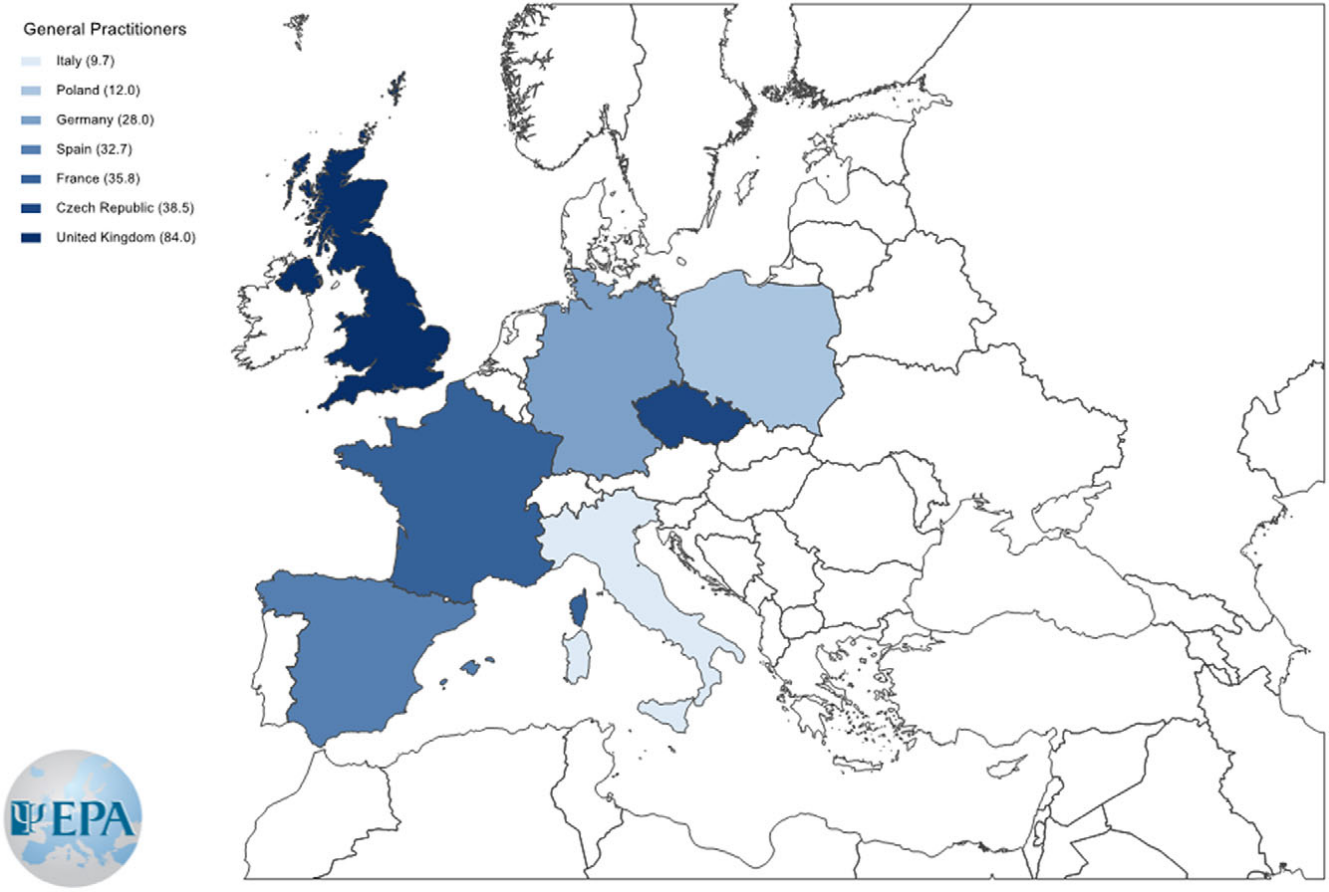
Color-coded map with a prevalence of general practitioners at the initiation of the eating disorder pathways to care in each participating European country.

**Figure 3.**
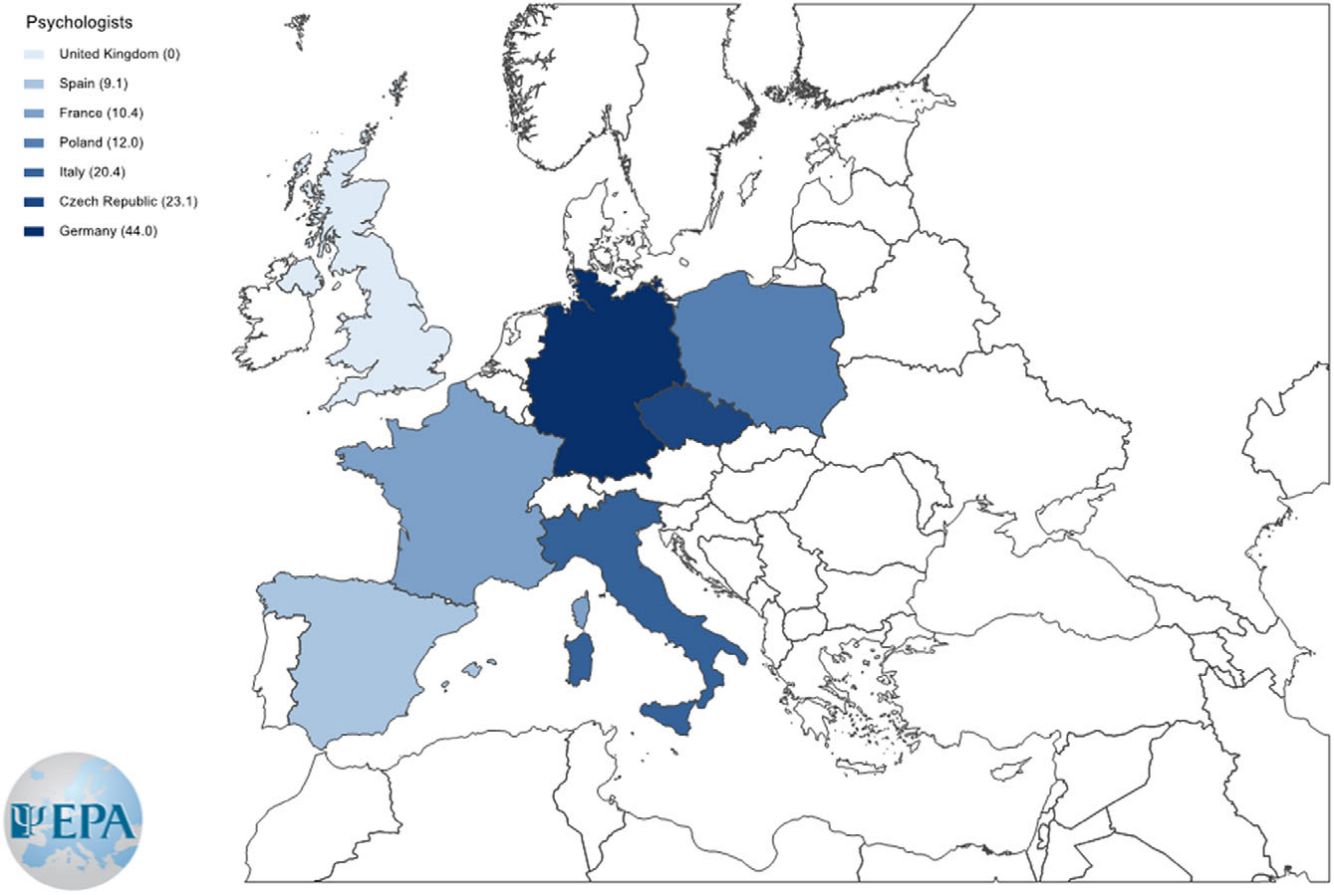
Color-coded map with a prevalence of psychologists at the initiation of the eating disorder pathways to care in each participating European country.

- *Median time elapsing between the onset of symptoms of the current ED episode and the access to a specialist ED unit.* Spain showed a delayed access of patients to ED units in comparison to all the other countries: these differences remained significant (*p <*0.001) after Bonferroni correction in the comparison with Poland, Italy, and UK. The same findings were observed in terms of the *length of the pathway* (namely, the months elapsing between access to the first health professional of the pathway and the access to the ED unit).
- *Number of health professionals. The* Czech Republic and Germany reported a higher (*p <*0.001) number of *health professionals* in the ED pathway to care than the other European countries.
- *Health professionals who were seen before the access to the ED unit:* general practitioners were less frequent in Italy (11.8%) than in France (35.8%), Spain (29.1%), and UK (68%). The prevalence of general practitioners was higher in UK (68%) than in the other countries. No differences among countries emerged for psychiatrists, while psychologists were more common in Germany (60%) than in other countries except Poland (20%) and Czech Republic (34.6%).
- *First health professionals of the pathway to specialist ED care.* General practitioners were less frequent in Italy (9.7%) and more frequent in UK (84%) than in other European countries (Figure 2). No differences among countries emerged for psychiatrists, while psychologists were more common in Germany (44%) than in the other countries (Figure 3).
- *Symptoms that promoted the arrival at the specialist ED unit.* Eating-related symptoms were less common in France (62.5%) than in Czech Republic (100%), Italy (91.4%), Spain (98.2%), and Germany (87.7%). General psychiatric symptoms were more common in Czech Republic (100%) than in Poland (60%), Italy (57.5%), UK (32%), and Germany (48%). In UK general psychiatric symptoms were less common than in France (80%) and Spain (80%). Somatic symptoms were more frequent in Czech Republic (88.5%) and less frequent in Germany (4%) and in UK (4%) than in other countries.
- *Suggestion to seek care at the specialist ED unit.* In Poland families and partners (88%) were more involved than in other European countries, while health professionals (4%) were less involved than in France (40.3%) and Spain (43.6%) and patients themselves sought care less frequently in Poland (24%) than in Spain (67.3%).

### Predictors of the length of the pathway to care

Longer time between the onset of symptoms of the current ED episode and access to a specialist ED unit was associated with treatment in a non-Italian center (HR 0.69, *p =* 0.01), higher age (HR 0.90, *p <*0.001), general psychiatric symptoms (HR 0.69, *p =* 0.01), low social class (HR 0.58, *p =* 0.008), suggestion to seek help by friends (HR 0.37, *p =* 0.019) or by health professionals (HR 0.49, *p <*0.001). The occurrence of somatic symptoms was associated with a shorter time from symptom onset to referral to the ED unit (HR 1.51, *p =* 0.004). Among non-Italian centers, Spain (HR 0.46, *p <*0.001), Czech Republic (HR 0.38, *p <*0.001), and Germany (HR 0.40, *p =* 0.002) were associated with delayed access to ED units, while UK had an earlier access (HR 1.91, *p =* 0.017).

## Discussion

This is a multicenter study exploring the specialist pathway to ED care in European countries. Most patients reached a specialist ED unit after seeing two other health professionals. Psychiatrists, general practitioners, and psychologists were the most common health professionals who either started the specialist pathway to ED care or directly referred to the specialist ED unit. Eating, depressive, anxious, and somatic symptoms promoted the activation of a specialist pathway to ED care and were even more common at the referral to the ED unit. The suggestion to seek care at a specialized ED unit came most frequently from relatives and patients themselves. Pathway-related variables (namely, suggestion to seek care by friends or by health professionals and the occurrence of general psychiatric symptoms that promoted seeking care), higher age, and low social class predicted a delayed access to the ED unit.

The median time between ED symptoms onset and access to a specialist ED unit was 2 years. This is in line with findings from previous Italian pathways to ED care study [29] and with international findings [10]. Most (92%) patients did not directly access a specialist ED unit and usually saw two other health professionals first. Only one out of four patients consulted a psychiatrist before seeking help at a specialist ED unit in spite of the central role that psychiatrists should play in the multidisciplinary treatment of EDs [18]. Along with psychiatrists and psychologists, general practitioners were among the most frequent health professionals in the ED pathway. This corroborates the importance of non-mental health professionals in the early diagnosis of EDs and in their referral to specialist care [23]. However, data from the literature reveal low rates of recognition for AN and BN by specialized medical professionals [22, 23] and general practitioners [38–40]. The involvement of general practitioners in the ED pathways was less frequent in Italy and more common in UK than in the other countries, while psychologists were most frequently involved in the pathway in Germany. These differences may reflect the local organization of health services, as in UK general practitioners are gate keepers of access to specialist care and most specialist ED services do not accept self-referrals.

The most frequent symptoms occurring when patients asked for help were eating-related, anxious, depressive, and somatic symptoms. This is in line with the high rate of comorbid affective disorders in individuals with EDs [41] and with the high relevance of these symptoms in the ED psychopathology [42–44]. General psychiatric symptoms were associated with a delayed access to ED unit, whereas somatic symptoms reduced the time to specialist ED care. The presence of other than EDspecific symptoms may prompt people to seek help more broadly and this aligns with the high rate of medical hospitalizations [22] and of central nervous system and gastrointestinal drugs prescriptions [23] received during the two years before the ED diagnosis. Interestingly, a notable proportion of patients also sought care to feel less depressed or less overwhelmed by emotional problems [45, 46]. This may be because there is shame or fear of being stigmatized for the ED, or the anosognosia that is often reported associated with many aspects of the illness [12]. Indeed, individuals with EDs often deny having a problem or are reluctant to disclose information about their eating behaviors [46, 47].

The role of others in prompting individuals to seek care is nuanced. Prompting from friends and workmates was associated with a delayed access to ED units whilst prompting from family members and partners with a shortened access time. While previous studies have suggested a facilitator role for a wider group of social networks including friends and peers [11, 48], we found a specific role of family members and partners, who seem to play a more active role in likely encouraging the individuals to seek help in the entire course of ED treatment [49, 50].

Two further factors were associated with the length of the ED pathway. First, the country where the treatment was conducted: Spain, Czech Republic, and Germany were associated with delayed access to ED units in comparison to Italy, while UK had an earlier access. These findings are in line with the heightened length of the pathway in Spain and the higher number of health professionals included in the pathway in Germany and may reflect differences in the organization of healthcare systems among European countries [51]. Second, belonging to a lower social class was a barrier to ED unit access: this may be due to the cost of treatment, or to the inaccurate stereotypes that are held about EDs that they are a problem only in the higher social classes [52].

The main strength of this study is the use of a standardized and quantitative method to assess the pathways to care for EDs, while previous research was mainly focused on personal barriers to treatment (i.e. denial of the illness, treatment perception or stigma problems), consisted of qualitative studies and used not-validated instruments [11, 12]. Also, this is the first study to assess the strength of the relationships between the various treatment barriers and the delay in the access to specialist treatment. Future studies which focus on denial of illness, stigma towards EDs, severity and type of symptoms assessed through standardized tools, perception [53], and cost of treatment or time spent in waitlist might be relevant [54].

Some limitations of the study need to be acknowledged. First, the recruiting centers did not cover the entire Europe zone, limiting generalizability. Second, some of the study findings may be at least partially affected by the organization of ED services and of the healthcare systems that can differ between European countries. Third, the patients were enrolled after the first wave of COVID-19 pandemic restrictions, and this may have affected the study results.

Clinical implications include increasing clinical competence of health care providers early in the care pathway, primarily general practitioners who were more frequently seen at the start of the specialist pathway than before the access to the ED unit. Brief screening measures that do not focus on ED symptoms alone but also on broader affective and somatic symptoms may be useful. Second, social media campaigns or school-based education programs [55] may turn to family members as playing a key role in prompting care seeking and need to be informed about warning signs of EDs pathways to refer their loved one to specialist ED care. Third, governments and politicians should turn greater attention to patients belonging to lower social classes, whose access to ED units seems delayed, and health professionals need to be educated about the links between low socio-economical states and EDs. These suggestions may help to improve public health interventions. Indeed, a recent public intervention was not effective in shortening the duration of untreated illness and the time to the first contact with health care professionals, although the authors suggested that the failure was due to methodological limitations (i.e., the intervention did not achieve the target group, or the exposure period was not sufficient) [56]. Therefore, current findings point to the need for a more tailored target of public health interventions.

To conclude, this multicenter European study suggests health care system organization that general practitioners, psychiatrists and psychologists, and family members play an important role in the early access to specific ED treatment. Affective and somatic symptoms are important in care seeking. If confirmed by future studies, these findings may contribute to developing educational programs that may help to increase the awareness of EDs and reduce delays in accessing specialist ED services.

## Supplementary material

For supplementary material accompanying this paper visit http://doi.org/10.1192/j.eurpsy.2023.23. click here to view supplementary material
