# Supplementary material for: Pathways to eating disorder care: A European multicenter study
Source: Eur Psychiatry. 2023 Apr 24;66(1):e36. doi: 10.1192/j.eurpsy.2023.23 (PMC10228357; doi:10.1192/j.eurpsy.2023.23)
Supplement: Supplementary file 1 [file S0924933823000238sup001.docx]

**Supplementary material**

*Methods*

The “Encounter Form” (EF) (1) adapted to the Italian organization of specialist eating disorder (ED) units (2) was employed. The EF allows researchers to collect, for each recruited patient, the following variables: demographic data, socio-economic status (as assessed referring to the Barratt Simplified Measure of Social Status (3)), the health professionals who were included in the pathway to specialized ED units, the main psychiatric problem(s) and the occurrence of eating-related symptoms leading the patient to seek treatment, who suggested the patient to seek care, the kind of treatment offered by the ED unit and the time interval between the onset of symptoms of the current ED episode and the admission to the specialist ED unit. The eating specific and the general psychological problems which favored the seeking care were not mutually exclusive: multiple symptoms may have been identified as promoting the referral. A current episode is defined as any ED episode for which the patient has not been immediately/directly referred to a specialist ED unit from another ED unit. The age of onset of the current ED episode is defined as the age when the patient first met DSM-5 diagnostic criteria for the current ED episode.

With respect to the Italian version of the EF (2), the modified EF employed in this study included the following items: age of onset of current ED episode; whether a history of care by any ED unit occurred and at what age and how long this treatment lasted; the kind of psychotherapy carried out at each ED unit. The EF took between 5 and 10 minutes to be completed.

Each PI participating in the research was provided with an instruction and coding Use the "Insert Citation" button to add citations to this document.

manual. If patients were not fully able to supply reliable information about the timing of each care step or the timing of symptoms onset, information was retrieved from family members or previous therapists.

**References**

1. Gater R, Sousa DBAE, Barrientos G, Caraveo J, Chandrashekar CR, Dhadphale M, et al. The pathways to psychiatric care: a cross-cultural study. Psychol Med. 1991;21(3):761–74.

2. Volpe U, Monteleone AM, Ricca V, Corsi E, Favaro A, Santonastaso P, et al. Pathways to specialist care for eating disorders: An Italian multicentre study. European Eating Disorders Review. 2019;27(3).

3. Barratt W. The Barratt simplified measure of social status (BSMSS). . Indiana State University. 2006;629.
